# Hydrocellular Foam Dressing Promotes Wound Healing along with Increases in Hyaluronan Synthase 3 and PPARα Gene Expression in Epidermis

**DOI:** 10.1371/journal.pone.0073988

**Published:** 2013-08-22

**Authors:** Takumi Yamane, Gojiro Nakagami, Sawako Yoshino, Aimi Muramatsu, Sho Matsui, Yuichi Oishi, Toshiki Kanazawa, Takeo Minematsu, Hiromi Sanada

**Affiliations:** 1 Department of Gerontological Nursing/Wound Care Management, Graduate School of Medicine, the University of Tokyo, Tokyo, Japan; 2 Department of Nutritional Sciences, Faculty of Applied Bioscience, Tokyo University of Agriculture, Tokyo, Japan; MGH, MMS, United States of America

## Abstract

**Background:**

Hydrocellular foam dressing, modern wound dressing, induces moist wound environment and promotes wound healing: however, the regulatory mechanisms responsible for these effects are poorly understood. This study was aimed to reveal the effect of hydrocellular foam dressing on hyaluronan, which has been shown to have positive effects on wound healing, and examined its regulatory mechanisms in rat skin.

**Methodology/Principal Findings:**

We created two full-thickness wounds on the dorsolateral skin of rats. Each wound was covered with either a hydrocellular foam dressing or a film dressing and hyaluronan levels in the periwound skin was measured. We also investigated the mechanism by which the hydrocellular foam dressing regulates hyaluronan production by measuring the gene expression of *hyaluronan synthase 3* (*Has3*), *peroxisome proliferator-activated receptor* α (*PPARα*), and *CD44*. Hydrocellular foam dressing promoted wound healing and upregulated hyaluronan synthesis, along with an increase in the mRNA levels of *Has3*, which plays a primary role in hyaluronan synthesis in epidermis. In addition, hydrocellular foam dressing enhanced the mRNA levels of *PPARα*, which upregulates *Has3* gene expression, and the major hyaluronan receptor *CD44*.

**Conclusions/Significance:**

These findings suggests that hydrocellular foam dressing may be beneficial for wound healing along with increases in hyaluronan synthase 3 and PPARα gene expression in epidermis. We believe that the present study would contribute to the elucidation of the mechanisms underlying the effects of hydrocellular foam dressing-induced moist environment on wound healing and practice evidence-based wound care.

## Introduction

In the past, it was generally accepted that wounds should be kept as dry as possible. During the 1960s, however, wound care took a new direction with the widespread introduction of the concept of moist wound healing [1]. Development and spread of a wound dressing made this possible in clinical practice. The use of occlusive dressings has been shown to accelerate wound healing. Occlusive dressings are reported to improve migration of keratinocytes over the moist wound surface instead of under the scab, and promote epithelialization associated with increased precipitation of fibrinogen and fibronectin [2,3]. A hydrocellular foam dressing has recently became a first choice for the treatment of moderately to heavily exuding wounds which require excessive wound debridement for providing optimum conditions at the wound site while maintaining a moist wound environment [4]. Wound exudate occurs due to bacterial infection, and the amount of wound exudate is directly proportional to the degree of bacterial colonization [5]. Excessive exudate, if allowed to remain on the wound bed, causes tissue maceration, which promotes slough formation and delay wound healing [5,6]. A hydrocellular foam dressing prevents this problematic situation. However, the molecular mechanism underlying a hydrocellular foam dressing-induced wound healing are poorly understood.

In this study, we propose a hypothesis regarding molecular mechanism responsible for hydrocellular foam dressing-induced wound healing, which focuses on hyaluronan, a major extracellular matrix component of the skin. Hyaluronan plays important roles in formation of scaffolds to promote tissue repair or regeneration, and biological functions such as cellular proliferation and migration at the time of wounding [7]. In mammals, hyaluronan is synthesized by isoforms of hyaluronan synthase (Has), namely, Has1, Has2, and Has3; these synthases have specific properties and are the products of distinct genes that differ in tissue distribution and regulation [8]. For example, Has2 is the major producer of hyaluronan in the dermis, whereas Has3 is the major hyaluronan producer in the epidermis. Has2 and Has3 mRNA expression are upregulated by peroxisome proliferator-activated receptor α (PPARα), that belongs to a family of nuclear receptors [9]. Hyaluronan mediates its biological effects through binding interactions with specific cell-associated receptors. CD44 is a polymorphic transmembrane glycoprotein that serves as the principal cell surface receptor for hyaluronan and can affect numerous cellular processes such as keratinocyte proliferation and migration at all stage of healing [10]. The migration of epidermal keratinocytes from the wound edge has been shown to promote wound healing [11]. We hypothesized that the polyurethane foam absorbs and retains the wound fluid, which might alter the biological behavior of the periwound skin to promote wound healing. Therefore, we focused on the relationship between hydrocellular foam dressing and hyaluronan synthesis.

In the present study, we used an animal model to examine the effect of hydrocellular foam dressing on hyaluronan, which has been shown to have positive effects on wound healing, and to investigate its molecular mechanisms. We measured hyaluronan levels in rat periwound skin by enzyme-linked immunosorbent assay (ELISA), and investigated the mechanism by which the hydrocellular foam dressing regulates hyaluronan production by measuring the gene expression of *Has3*, *PPARα*, *CD44* by using quantitative reverse transcription-polymerase chain reaction (RT-PCR).

## Materials and Methods

### Materials

A QnE hyaluronic acid ELISA assay was purchased from Biotech Trading Partners (Encinitas, CA, USA). ALLEVYN Non-Adhesive, a hydrocellular foam dressing consists of hydrophilic polyurethane and polyethylene glycol, was purchased from Smith & Nephew Medical Ltd. (Hull, UK). Tegaderm^TM^ HP transparent dressing, a film dressing, was purchased from 3M Health Care (St. Paul, MN, USA). The SV Total RNA Isolation System was purchased from Promega Corp. (Madison, WI, USA) and a High-Capacity cDNA Reverse Transcription Kit was obtained from Applied Biosystems (Foster City, IN, USA).

### Full-thickness cutaneous wound model

**Figure 1:**
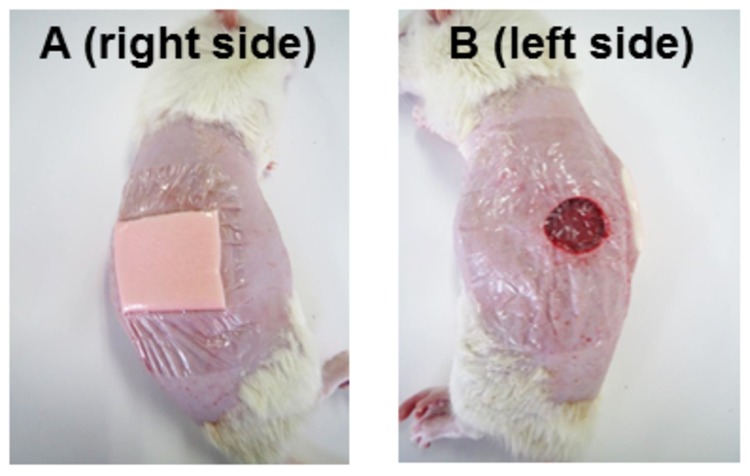
Full-thickness cutaneous wound model. Two full-thickness wounds, a diameter of 1.5 cm, were created in the dorsolateral skin of five rats using sterile scissors. Each wound was covered with either a hydrocellular foam dressing (A) or the film dressing (B). A film dressing was used as a secondary dressing in both sides of individual rat and also used to keep the hydrocellular foam dressing attached to the wound.

Male specific-pathogen-free 7-week-old Wistar rats were purchased from Japan SLC Inc. (Shizuoka, Japan). Animals were given food and ultrafiltered water ad libitum, and were maintained on a 12-h/12-h light/dark cycle. Two full-thickness wounds, a diameter of 1.5 cm, were created in the dorsolateral skin of five rats using sterile scissors, under sedation with an intraperitoneal injection of Somnopentyl (pentobarbital sodium; Kyoritsu Seiyaku Corporation, Tokyo, Japan) (30 mg/kg body weight). The subcutaneous fat layer was completely dissected to expose the fascia. Each wound was covered with either a hydrocellular foam dressing (Figure 1A) or the film dressing (Figure 1B). A film dressing was used as a secondary dressing in both sides of individual rat and also used to keep the hydrocellular foam dressing attached to the wound. The wound area was measured every day until 7 days after wounding using image analysis software (IMAGEJ version 1.42; NIH, Bethesda, MD), and the results were expressed relative to the initial wound area. Periwound skin samples (5 mm thick, taken from the edge of the wound) were collected on day 4 under anesthesia with an intraperitoneal injection of Somnopentyl (30 mg/kg body weight) and were subjected to quantification of hyaluronan or RNA preparation. The experimental protocol was approved by the Animal Research Committee of The University of Tokyo. All animals were treated according to the Guide for the Care and Use of Laboratory Animals of the NIH.

### Quantification hyaluronan

Skin samples were prepared as described previously [12]. Briefly, the skin pieces were defatted with acetone, dried, weighed, boiled for 20 min in 50 mM Tris/HCl (pH 7.8) buffer, and then subjected to proteolytic digestion with 1% w/v actinase E for a week at 40°C. Trichloroacetic acid was added to the samples at a final concentration of 10% w/v for deproteinization before centrifugation at 3000 rpm at 4°C for 20 min. The supernatants thus obtained were neutralized with 10 N NaOH. Hyaluronan levels were measured using the QnE hyaluronic acid ELISA, according to the manufacturer’s instructions. Properly diluted specimens and hyaluronan reference solutions are incubated in hyaluronic acid binding protein (HABP)-coated microwells. After the removal of unbound molecules by washing, HABP conjugated with horseradish peroxidase solution is added to the microwells to form complexes with bound hyaluronan. Following another washing step, a chromogenic substrate of tetramethylbenzidine and hydrogen peroxide is added to develop a colored reaction. The intensity of the color is measured in optical density units with a spectrophotometer at 450 nm. Hyaluronan levels in specimen samples and control samples are determined against a reference curve prepared from the reagent blank (0 ng/ml) and the hyaluronan reference solutions provided with the kit (50, 100, 200, 500, 800 ng/ml).

### RNA extraction and quantitative RT-PCR

Fresh and deep-frozen rat skin samples were separated at the dermo-epidermal basement membrane as described by Trost et al. [13]. Total RNA was extracted from the skin by using the SV Total RNA Isolation System, according to the manufacturer’s instructions. The concentration of RNA was determined by absorbance at 260 nm and the purity was indicated by 260/280 nm ratio with values consistently between 2.0 and 2.2. The integrity and quantification of RNA were confirmed by visualization of rRNAs after electrophoresis on denaturing agarose gel. Reverse transcription was performed using a High-capacity cDNA Reverse Transcription Kit under the following conditions: 37°C for 2 h and 85°C for 30 s after preheating at 25°C for 10 min. The amplification products obtained were detected using a SYBR Green PCR Master Mix and real-time RT-PCR (stratagene MX 3000P, Agilent Technologies, Japan). Amplifications were performed under the following conditions: 40 cycles of 95°C for 30 s and 60°C for 1 min after preheating at 95°C for 10 min. The relative expression levels of each target gene were calculated using the C_t_ method using glyceraldehyde-3-phosphate dehydrogenase (GAPDH) gene as an internal control. The sequences of the Has3, PPARα, CD44 and GAPDH primers were as follows: Has3 forward, 5’-GTGTTCGAGCTGTGGTGTGG-3’ and Has3 reverse, 5’-GGGGATCTTCCTCCAAGACC-3’ (GeneBank accession no: NM_172319.1) ; PPARα forward, 5’-TGAACAAAGACGGGATG-3’ and PPARα reverse, 5’-TCAAACTTGGGTTCCATGAT-3’ (GeneBank accession no: NM_013196.1) ; CD44 forward, 5’-CCGTTACGCAGGTGTATTCC-3’ and CD44 reverse, 5’-TGTTGAAAGCCTCGCAGAG-3’ (GeneBank accession no: NM_012924.2) ; GAPDH forward, 5’-TGGTGAAGGTCGGTGTGAAC-3’ and GAPDH reverse, 5’-GACTGTGCCGTTGAACTTGC-3’ (GeneBank accession no: NM_017008.4).

### Statistical analysis

Results are expressed as mean ± SE. Repeated measures analysis of covariance with Tukey’s multiple comparison was performed at each time point to compare the differences between the side of film dressing and hydrocellular foam dressing. Statistical differences between two groups were determined using the Student’s *t*-test. The statistical significance level was set at *p* < 0.05.

## Results

### Hydrocellular foam dressing promoted wound healing in rat skin

**Figure 2:**
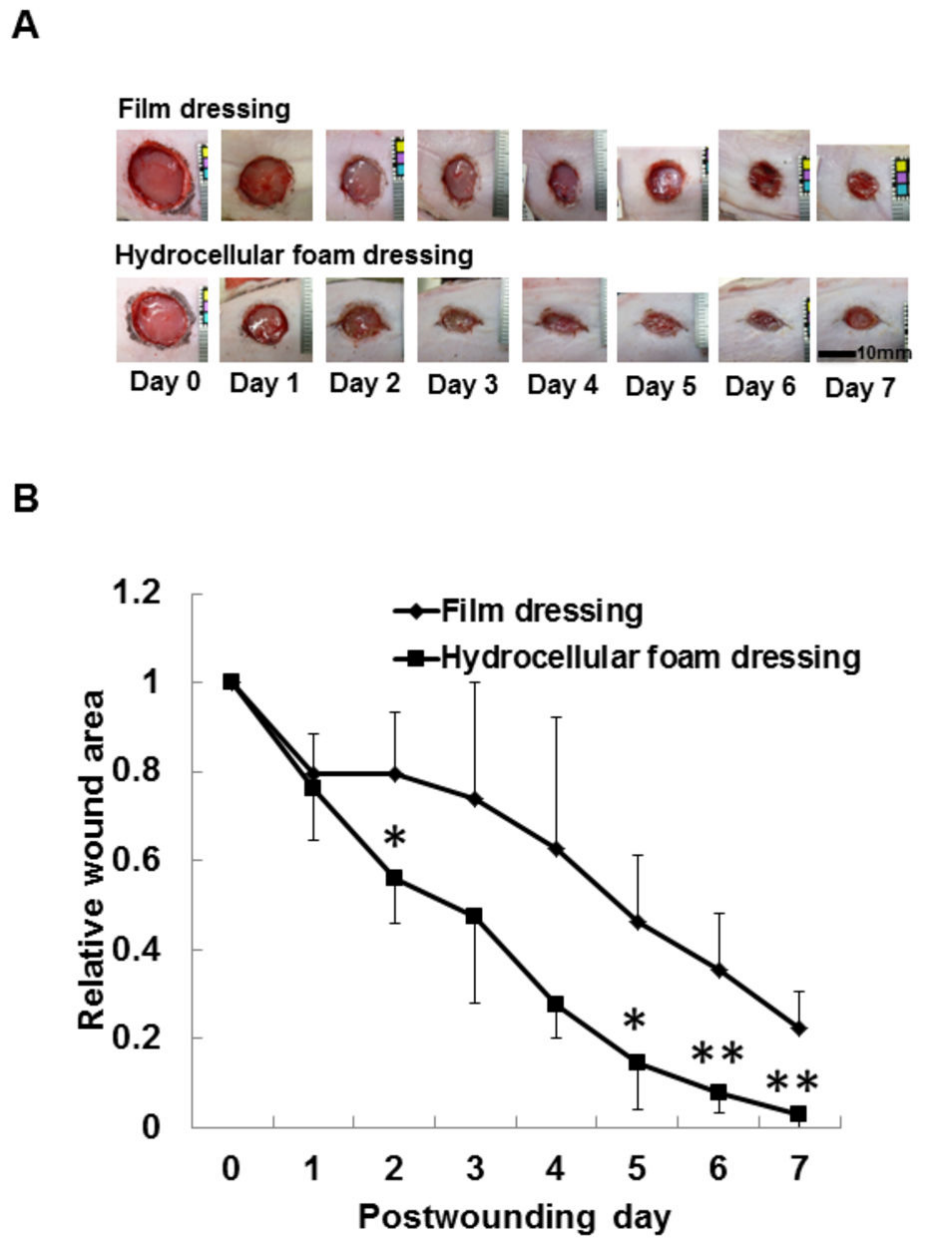
Effects of hydrocellular foam dressing on wound healing in rat skin. Gross observations (A) and the relative wound areas (B) reveal increased wound contraction induced by hydrocellular foam dressing. Bars are expressed as mean ± SE (n = 4). *p < 0.05 indicates values that are significantly different from the side of film dressing in rat skin.

The time-course effect of hydrocellular foam dressing on wound healing in rat skin is shown in Figure 2A and B. Gross observations revealed increased wound contraction and promoted re-epithelialization of wounds treated with hydrocellular foam dressings, compared with those treated with film dressings (Figure 2A). We detected significant differences in wound size at day 2 and days 5-7 post-wounding (Figure 2B).

### Effects of hydrocellular foam dressing on hyaluronan level and Has3 mRNA expression in periwound epidermis

**Figure 3:**
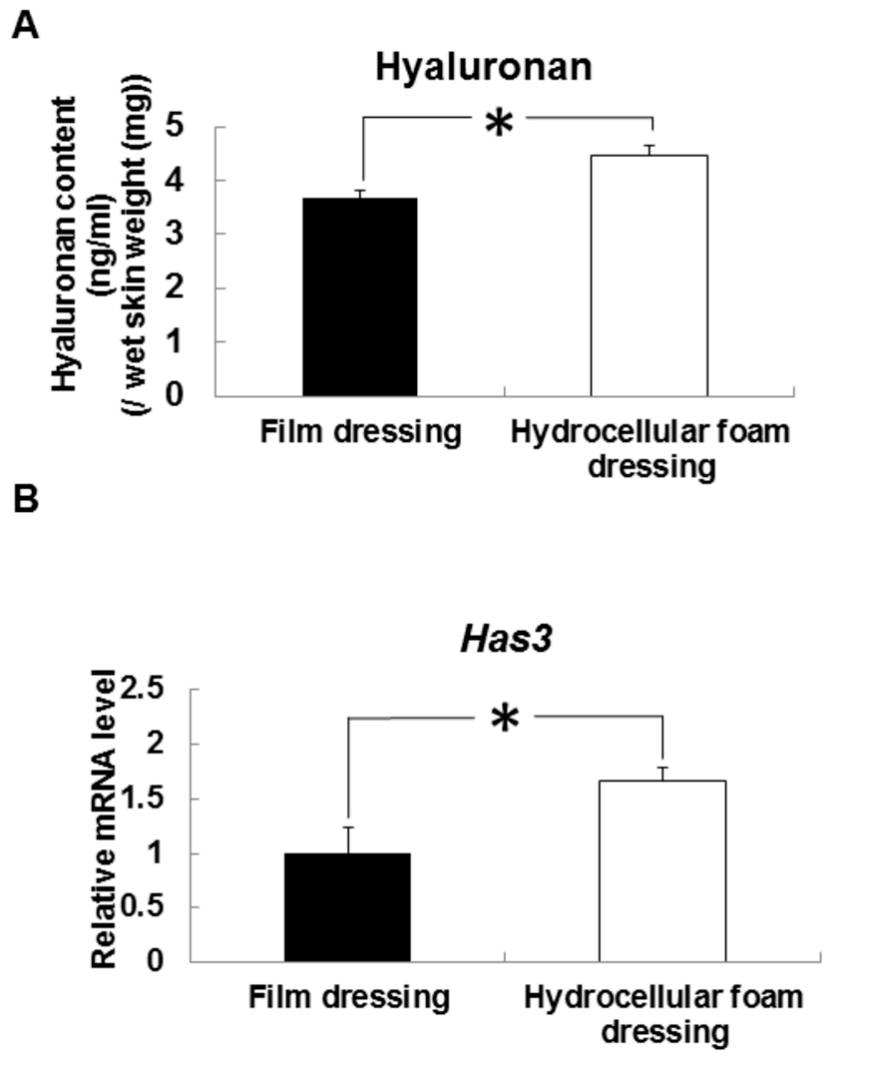
Effects of hydrocellular foam dressing on level of hyaluronan and Has3 mRNA expression in periwound epidermis. Hyaluronan levels in periwound epidermis (A) were measured using a QnE hyaluronic acid ELISA assay. Has3 mRNA levels in periwound epidermis (B) were measured by quantitative PCR and expressed as values relative to those of GAPDH. Bars are expressed as mean ± SE (n = 4, 5). *p < 0.05 indicates values that are significantly different from the side of film dressing in rat skin.

The effect of hydrocellular foam dressing on hyaluronan synthesis in rat skin was investigated. Hyaluronan levels were significantly increased in the epidermis around the wounds covered with hydrocellular foam dressings, compared with those covered with film dressings (Figure 3A). We also investigated Has3 mRNA levels in the peri-wound epidermis and found significantly higher levels of Has3 mRNA around wounds covered with hydrocellular foam dressings, compared with those covered with film dressings (Figure 3B).

### Effects of hydrocellular foam dressing on the level of PPARα mRNA expression in periwound epidermis

**Figure 4:**
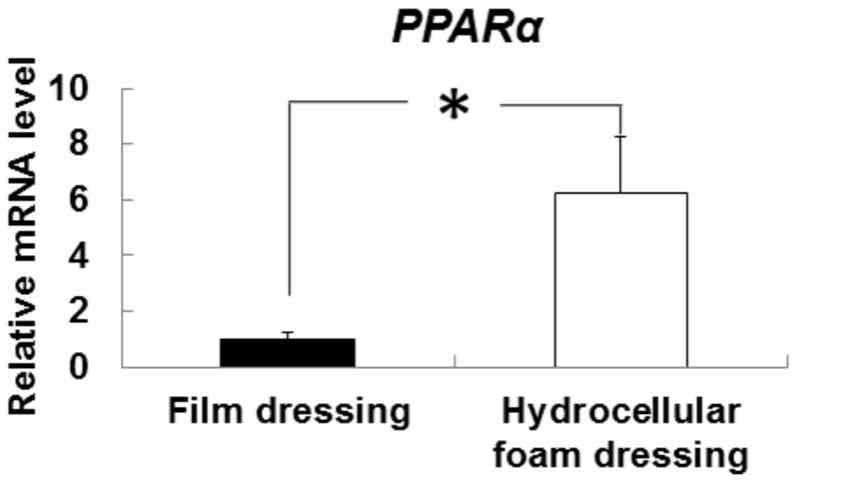
Effects of hydrocellular foam dressing on PPARα mRNA levels in periwound epidermis. The PPARα mRNA levels in periwound epidermis were measured by quantitative PCR and expressed as values relative to those of GAPDH. Bars are expressed as mean ± SE (n = 5). *p < 0.05 indicates values that are significantly different from the side of film dressing in rat skin.

Our preliminary experiments showed that PPARα agonist WY14,643 enhanced Has3 mRNA expression in human keratinocytes (data not shown). We therefore investigated PPARα mRNA levels in epidermis around the wound (Figure 4). PPARα mRNA levels were significantly higher around wounds covered with hydrocellular foam dressings, compared with those covered with film dressings.

### Effects of hydrocellular foam dressing on CD44 mRNA expression in periwound epidermis

**Figure 5:**
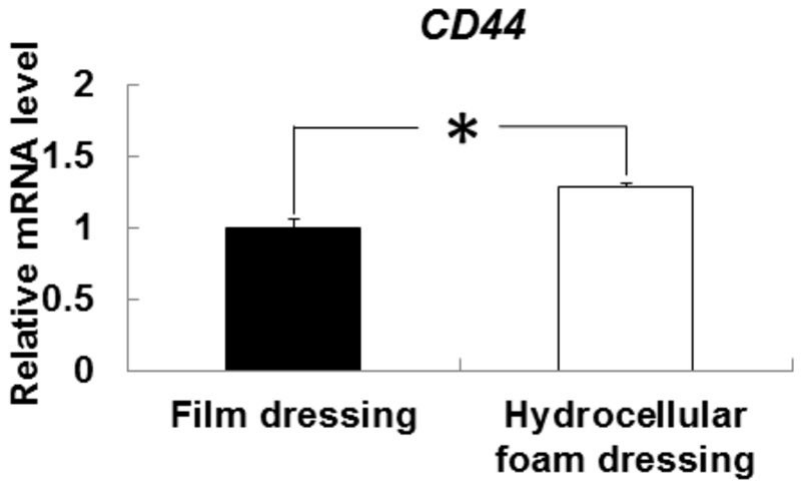
Effects of hydrocellular foam dressing on CD44 mRNA levels in periwound epidermis. CD44 mRNA levels in periwound epidermis were measured by quantitative PCR and expressed as values relative to those of GAPDH. Bars are expressed as mean ± SE (n = 5). *p < 0.05 indicates values that are significantly different from the side of film dressing in rat skin.

We investigated the mRNA levels of CD44, which is the major receptor for hyaluronan on the surface of epidermal keratinocytes (Figure 5). CD44 mRNA levels were significantly higher around the wounds covered with hydrocellular foam dressings, compared to those covered with film dressings.

## Discussion

Hydrocellular foam dressing-induced moist environment has been reported to promote wound healing [14]; however, the molecular mechanisms responsible for these effects remain unknown. The present study therefore examined the effects of hydrocellular foam dressing on hyaluronan, which has been shown to have positive effects on wound healing, and on the regulatory mechanisms responsible for these effects. The results provide the first evidence that hydrocellular foam dressings increase mRNA levels of Has3 and PPARα, which contribute to wound healing, along with increases in hyaluronan synthesis in periwound skin.

Hyaluronan is a fundamental component of the extracellular matrix and plays crucial roles in cellular migration, proliferation and differentiation. Has3 is the major producer of hyaluronan in the epidermis. Tammi et al. [15] suggested that epidermal injury upregulates the expression of Has3 in keratinocyte and that hyaluronan synthesis has an important role in the re-epithelialization, which involves keratinocyte migration. Our data showed that hydrocellular foam dressing promoted wound healing and the level of hyaluronan along with increases in Has3 transcripts. Therefore, it is likely that hydrocellular foam dressing promotes wound healing by increasing hyaluronan synthesis, which contributes to keratinocyte proliferation and migration in rat skin around the wound.

To clarify the mechanisms for the upregulation of Has3 mRNA expression in the epidermis around the wounds covered with hydrocellular foam dressings, we focused on the action of PPARα, which is a transcription factor. PPARα have been shown to be involved in keratinocytes differentiation, prolifelation and epidermal wound repair during the different phases of the healing process [16]. Kim et al. [9] reported that WY14,643, a PPARα agonist, promoted hyaluronan synthesis along with increases in HAS3 transcripts in human dermal fibroblasts. Furthermore, we observed that PPARα agonist promoted Has3 mRNA expression in human keratinocytes (data not shown). In the present study, hydrocellular foam dressings enhanced the PPARα mRNA levels in epidermis around the wound. Changes in cytokine and/or growth factor levels at wound surface may contribute to promotion of PPARα gene expression because hydrocellular foam dressings cause concentration and component changes of wound fluid at wound surface by absorbing fluid. Fluid taken from acute wounds contains pro-inflammatory cytokines such as tumor necrosis factor α (TNF-α) and interleukin 1β (IL-1β) [17]. Fang et al. [18] reported that TNF-α and IL-1β suppress PPARα expression and activity via activation of the nuclear factor-kappa B pathway in hepatocytes. Thus, changes in TNF-α and IL-1β levels at wound surface may contribute to promotion of PPARα gene expression by hydrocellular foam dressings. Taken together, we firstly propose a possibility that the promotion of wound healing by hydrocellular foam dressings may partially result from increased hyaluronan synthesis due to the action of PPARα.

Hyaluronan induces intracellular signals through binding to cell surface receptor such as CD44, which contributes to keratinocyte proliferation and migration and the maintenance of local hyaluronate homeostasis. Luo et al. [19] reported that downstream signaling of CD44 promoted cellular proliferation through the activation of the mitogen-activated protein kinase pathway in human keratinocytes. The present study showed that hydrocellular foam dressing promoted CD44 mRNA expression in epidermis around the wound. CD44 is highly expressed in migrating keratinocytes at all stage of healing, and hyaluronan-mediated CD44 activation upregulates wound healing [10,20]. CD44, via its interaction with hyaluronan, should thus be considered as a potent regulator of wound healing under hydrocellular foam dressing-induced moist conditions. However, further studies are needed to establish the precise mechanism underlying the increase in CD44 gene expression.

Our study focused on the effect of hydrocellular foam dressing on the periwound skin, especially for on the role of hyaluronan production in the epidermis. To clarify the mechanisms resposible for the wound-promoting effect of this type of dressing, the other stages of wound healing, such as granulation tissue formation phase, should be addressed. Kunugiza et al. [21] reported that the hydrocellular foam dressings accelerated neovascularization in the granulation tissue by enhancing vascular endothelial growth factor. Taken together, this type of dressing has beneficial effects on both granulation tissue and re-epithelialization in the full-thickness wound.

In summary, we have shown that hydrocellular foam dressing may be beneficial for wound healing along with increases in hyaluronan synthesis in epidermis. This study provides the first evidence for an effects of hydrocellular foam dressings on Has3 and PPARα gene expression, leading to the promotion of hyaluronan synthesis in the periwound skin. Although our results did not support the direct evidence for involvement of PPARα, this study proposed the possibility that a wound dressing containing a PPARα agonist may be an advanced treatment method for enhancing the body’s natural healing ability through increasing in Has3 expression. We believe that the present study would contribute to the elucidation of the mechanisms underlying the effects of hydrocellular foam dressing-induced moist environment on wound healing and practice evidence-based wound care.
